# Connection of Turbulence with Polytropic Index in the Solar Wind Proton Plasma

**DOI:** 10.3390/e21111041

**Published:** 2019-10-25

**Authors:** George Livadiotis

**Affiliations:** Division of Space Science and Engineering, Southwest Research Institute, San Antonio, TX 78238, USA; glivadiotis@swri.edu; Tel.: +1-210-522-3415

**Keywords:** polytropic index, turbulence, solar wind, space plasmas

## Abstract

This paper improves our understanding of the interplay of the proton plasma turbulent heating sources of the expanding solar wind in the heliosphere. Evidence is shown of the connections between the polytropic index, the rate of the heat absorbed by the solar wind, and the rate of change of the turbulent energy, which heats the solar wind in the inner and outer heliosphere. In particular, we: (i) show the theoretical connection of the rate of a heat source, such as the turbulent energy, with the polytropic index and the thermodynamic process; (ii) calculate the effect of the pick-up protons in the total proton temperature and the relationship connecting the rate of heating with the polytropic index; (iii) derive the radial profiles of the solar wind heating in the outer and inner heliosphere; and (iv) use the radial profile of the turbulent energy in the solar wind proton plasma in the heliosphere, in order to show its connection with the radial profiles of the polytropic index and the heating of the solar wind.

## 1. Introduction

Solar wind protons flow throughout the supersonic heliosphere under expansive cooling and turbulent heating. The turbulence in solar wind has two sources: (i) the solar-origin large-scale energy fluctuations, and (ii) the excitation of plasma waves by newborn interstellar pickup ions. Other secondary sources may exist, e.g., turbulence can also be generated in-situ in the solar wind by velocity shears [1,2]. The turbulence affects the polytropic behavior of the solar wind plasma.

The meaning of the polytrope has its origin in the many turns of the thermodynamic state of a system. It stands for a family of thermodynamic processes that follow a specific relationship among thermal observables, e.g., density *n*, temperature *T*, and thermal pressure *p*, that is,

(1a) p(R→)∝n(R→)​ γ,​ or​ n(R→)∝T(R→)ν,

where the polytropic indices *γ* and *ν* are independent of the position vector, while they are related to each other with:

(1b) γ=1+1/ν​ or​ ν=1/(γ−1).

(Hereafter, the position vector $\vec{R}$ measures the heliocentric distance.) Frequently, space and astrophysical plasmas present positive correlations between density and temperature, caused by this polytropic relationship, with a polytropic index ranging between the values of the isothermal (*γ* = 1) and isochoric (*γ*→+∞) processes. The polytropic index of solar wind near 1 AU is close to the value of the adiabatic process (*γ* = 5/3) (e.g., see: [3,4,5,6,7,8,9,10,11,12]). Note that values of polytropic index smaller than the adiabatic value of 5/3 are used to account for unspecified heating mechanisms, such as turbulent heating. However, in a solar wind model with appropriate turbulence modeling, the actual heating mechanism is included in a more complete way, and in that case the natural value of *γ* = 5/3 can be used (e.g., [13]).

The solar wind is expected to expand into the heliosphere with almost no energy variation, and thus, it is cooled by an adiabatic expansion. However, the existence of turbulence significantly changes this ideal picture; the turbulent heating of the solar wind leads to a polytropic index smaller than its adiabatic value.

The solar wind plasma in the outer heliosphere is characterized by a polytropic index that slowly decreases with the heliocentric distance *R* [14]. On the other hand, the solar wind plasma in the inner heliosphere does not exhibit clear dependence of the adiabatic process with distance, though it is expected to have a slight decrease with *R* [15]. In any case, the inner heliospheric plasma is characterized by a variation of the polytropic index from its adiabatic value with *δ_γ_* ~ 0.2, that is, from a near adiabatic index *γ* ~ 1.65 (e.g., [9]), to some smaller value, e.g., *γ* ~ 1.45 (e.g., as derived from [16]).

Since the turbulent heating affects the solar wind thermodynamic process and polytropic index value, we may ask: Are the radial profiles of polytropic index *γ*(*R*) and the rate of change of the turbulent energy *dE*_t_/*dt* related to each other? Can we deduce the radial profile of turbulence rate from the knowledge of the radial profile of the polytropic index?

The purpose of this paper is to show the connection between the polytropic index and the turbulent energy rate in the solar wind proton plasma, by comparing their corresponding radial profiles in the outer and inner heliosphere. In Section 2, we show the theoretical connection of the rate of a heat source, such as the turbulent energy, with the polytropic index and the thermodynamic process. In Section 3, we calculate the effect of the pick-up protons in the total proton temperature and the relationship connecting the rate of heating with the polytropic index. In Section 4, we use the radial profile of the turbulent energy in the solar wind proton plasma in the outer and inner heliosphere. We show the connection between the radial profiles of the polytropic index and the turbulent heating of the solar wind in the outer and inner heliosphere. Finally, in Section 5, we summarize the results.

## 2. Thermodynamics of Polytropes

In this section, we briefly derive the expression for the temperature and heating evolution of the solar wind protons, considering polytropic radial expansion (see also: [17,18,19,20]). Given the polytropic $T\propto n^{\gamma -1}$ and the spherical expansion $n\propto R^{-2}$ relationships, we obtain $T\propto R^{-2(\gamma -1)}$, which is equivalent to the gradient equation:

(2) $$T\propto R^{-2(\gamma -1)}\Leftrightarrow dT/dR=-2(\gamma -1)\cdot T/R.$$

According to the first law of thermodynamics, in the absence of any energy exchange with the environment, the system is under an adiabatic process and the polytropic index is *γ*_a_ = 1 + 2/*D*, that is, *γ*_a_ = 5/3, for degrees of freedom (d.o.f.) *D* = 3. However, in the presence of heating, the thermodynamic process is not adiabatic and is described by a polytropic index *γ ≠* *γ*_a_.

The first thermodynamic law gives:

(3a) dU+dW=dQ,

with:

(3b) dU=12D⋅N⋅kBdT​ and​ dW=pdV,

where *U* is the internal energy of the *N*-particle system, and *W* is its work. Then, taking into account that *pV* = *Nk*_B_*T*, we find ½·*D*·*Vdp* + (½·*D* + 1)·*pdV* = *dQ*, and dividing by ½·*D*·*pdV*, we arrive at:

(4a) $$\frac{dQ}{pdV}=\frac{\gamma_{a}-\gamma }{\gamma_{a}-1},$$

or:

(4b) $$\gamma =1+(\gamma_{a}-1)\left(\frac{dQ}{pdV}-1\right),$$

where we used the definition of the polytropic index *d*ln*p/d*ln*V* = −*γ* (because *p*∝*V*^−^*^γ^*). Then, substituting *γ* from Equation (4b) into Equation (2), we derive:

(5) $$dT/dR=-2(\gamma_{a}-1)\cdot T/R+(\gamma_{a}-1)\cdot [dQ/(pdV)]\cdot T/R.$$

For d.o.f. *D* = 3, we have *V* = (4π/3)·*R*^3^ (or *dV* = 4π·*R*^2^*dR*) and *γ_a_*− 1 = ⅔; also, we have *p* = *N*·*k*_B_*T*/*V* and the heating per particle *q* = *Q*/*N*; then, we find:

(6) $$dT/dR=-2(\gamma_{a}-1)\cdot T/R+k_{B}{}^{-1}dq/dR=-2(\gamma -1)\cdot T/R.$$

Therefore, the gradient of the heat absorbed by the solar wind, *dq*/*dR*, is given by:

(7) $$dq/dR=2(\gamma_{a}-\gamma )\cdot k_{B}T_{p}/R,$$

where we consider the proton plasma with temperature *T*_p_. In order to find the heating rate *dq*/*dt*, we consider the advection speed of the heating:

(8) $$dq/dt=u_{R}\cdot dq/dR,$$

hence, Equation (7) becomes:

(9a) $$dq/dt=2(\gamma_{a}-\gamma )\cdot u_{R}\cdot k_{B}T_{p}/R.$$

(Note that according to [17], the dissipation rate per mass is $\varepsilon =-\frac{3}{2}dq/dt$.) Then, using the notion of proton thermal speed $\theta_{p}=\sqrt{2k_{B}T_{p}/m_{p}}$, where *m*_p_ is the proton mass, we obtain:

(9b) (dq/dt)/mp=(γa−γ)⋅uR⋅θp​ 2/R.

The advection speed is:

(10) uR={Vsw​ advection​ at​ flow​ speed​ ,θp​ ​ ​ advection​ at​ thermal​ speed​ ,

depending on whether the turbulent heating is advected at the solar wind plasma flow *V*_sw_ or the thermal speed *θ*_p_. Note that advection at thermal speed, instead of the bulk flow speed, is possible as a limiting case, e.g., see references [21,22,23].

## 3. Effect of Pick-up Ions

Here, we add to Equation (9) the effect of pick-up ions (PUIs). According to [24], the PUI temperature is summed to the solar wind proton temperature, as follows: The total proton pressure sums the solar wind and pickup proton partial pressures, *P*_p,tot_ = *P*_p_+*P*_pui_, i.e., (*n*_p_+*n*_pui_)*k*_B_*T*_p,tot_ = *n*_p_*k*_B_*T*_p_ + *n*_pui_*k*_B_*T*_pui_. The total temperature, *T*_p,tot_, is derived from mixing solar wind and pickup protons, i.e.,

(11a) Tp,tot=(Tp+nrTpui)/(1+nr)​ with​ nr≡npui/np,

while:

(11b) θp,​ tot=2kBTp,tot/mp,​ θp=2kBTp/mp,​ and​ θpui=2kBTpui/mp,

are the respective thermal speeds, which are related according to:

(11c) θp,​ tot​ 2=(θp​ 2​ +​ nrθpui​ 2)/(1​ +​ nr).

From Table 1 in McComas et al. [24], we can easily derive the radial dependence of solar wind temperature *T*_p_ and density *n*_p_, as well as the PUI temperature *T*_pui_ and density *n*_pui_:

*T*_p_/[K] ≈ 0.9984·10^5^ × (*R*/[AU])^−0.74^, *n*_p_/[cm^−3^] ≈ 5.140 × (*R*/[AU])^−1.8^, (12a)

*T*_pui_/[K] ≈ 4.069·10^5^ × (*R*/[AU])^0.68^, *n*_pui_/[cm^−3^] ≈ 3.006·10^−3^ × (*R*/[AU])^−0.58^, (12b)

*n*_r_ ≡ *n*_pui_/*n*_p_ ≈ 5.847·10^−4^ × (*R*/[AU])^1.22^. (12c)

Note: the 1 AU values, included in the above formulae, were estimated as the geometric averages of the two 1 AU values, which can be estimated via the 30 AU and 90 AU datasets of Table 1 in [24].

The respective thermal speeds are given by:

*θ*_p_/[km/s] ≈ 40.60 × (*R*/[AU])^−0.37^, *θ*_pui_/[km/s] ≈ 82.96 × (*R*/[AU])^0.34^, (12d)

while the average solar wind flow speed is:

*V*_sw_ /[km/s] ≈ 517.26 × (*R*/[AU])^−0.083^. (12e)

Note: Equation (12e) was derived from the estimations of *V*_sw_ = 390 km/s at 30 AU and *V*_sw_ = 356 km/s at 90 AU, as given in [24].

Therefore, the presence of PUIs affects Equation (9a), replacing the solar wind proton temperature with the total proton temperature:

(13a) $$dq/dt=2(\gamma_{a}-\gamma )\cdot u_{R}\cdot k_{B}T_{p,\mathrm{tot}}/R,$$

or, using the thermal speed,

(13b) $$(dq/dt)/m_{p}=(\gamma_{a}-\gamma )\cdot u_{R}\cdot \theta_{p,\mathrm{tot}}^{2}/R,$$

while the advection speed is now given by:

(14) uR={Vsw​ ​ ​ ​ ​ ​ advection​ at​ solar​ wind​ flow​ speed​ ,θp​ ​ ​ ​ ​ ​ ​ ​ advection​ at​ solar​ wind​ thermal​ speed​ ,θp,tot​ ​ ​ advection​ at​ solar​ wind​ total​ thermal​ speed​ .

Finally, the above can be expressed in terms of the heliocentric distance, *R*:

(15a) 1mpq˙(R)=δγ(R)⋅uR(R)⋅θp,tot​ 2(R)/R,

with $\delta_{\gamma }\equiv \gamma_{a}-\gamma$, or:

(15b) $$\begin{array}{l} \frac{1}{m_{p}}\dot{q}(R)/[{\mathrm{km}}^{2}{/s}^{3}]=\delta_{\gamma }(R)\times \\ \left\{ \begin{array}{l} 5.699\cdot 10^{-3}\cdot {\left(R/[\mathrm{AU}]\right)}^{-1.823}\cdot \frac{1+2.383\cdot 10^{-3}\cdot {\left(R/[\mathrm{AU}]\right)}^{2.64}}{1+5.847\cdot 10^{-4}\cdot {\left(R/[\mathrm{AU}]\right)}^{1.22}}\\ 4.473\cdot 10^{-4}\cdot {\left(R/[\mathrm{AU}]\right)}^{2.11}\cdot \frac{1+2.383\cdot 10^{-3}\cdot {\left(R/[\mathrm{AU}]\right)}^{2.64}}{1+5.847\cdot 10^{-4}\cdot {\left(R/[\mathrm{AU}]\right)}^{1.22}}\\ 4.473\cdot 10^{-4}\cdot {\left(R/[\mathrm{AU}]\right)}^{2.11}\cdot {\left[\frac{1+2.383\cdot 10^{-3}\cdot {\left(R/[\mathrm{AU}]\right)}^{2.64}}{1+5.847\cdot 10^{-4}\cdot {\left(R/[\mathrm{AU}]\right)}^{1.22}}\right]}^{\frac{3}{2}}, \end{array}\right. \end{array}$$

depending on the advection speed, as shown in Equation (14).

## 4. Polytropic Index Versus Turbulent Energy

The turbulent energy, developed along the solar wind radial expansion, is given by *E*_t_ /*m*_p_ = *σ_z_*^2^, that is, the variance of the Elsässer vector variable ${\vec{Z}}_{+}$. The Elsässer variables [25] are defined by:

(16) Z→±≡V→sw±V→a,​ with​ V→a=B→/μ​ ρ,

where ${\vec{V}}_{a}$ is the Alfvén velocity, *ρ* is the mass density ≈ *m*_p_·*n*. The Elsässer vector variable ${\vec{Z}}_{+}$ {${\vec{Z}}_{-}$} corresponds to Alfvénic modes with an outward {inward} radial direction of propagation (in the solar wind frame).

The turbulent energy heats the solar wind, and thus, affects the value of the polytropic index, which may vary from near adiabatic *γ* ≈ *γ_a_* to a smaller value *γ* < *γ_a_*. When the polytropic index is smaller than the adiabatic value, *γ* < *γ_a_*, then the plasma is endothermic, i.e., a heat-absorbing system. If there was no turbulent heating of the solar wind, then the radial expansion of the solar wind would be purely adiabatic and the polytropic index equal to *γ* = *γ_a_*. Below, we explain the connection between the polytropic index and the turbulent heating separately for the inner and outer heliosphere:

In the inner heliosphere, the solar-origin large-scale energy fluctuations constitute the primary source of turbulent heating. This turbulent energy decreases with heliocentric distance as [26,27,28]:

*E*_t_ /*m*_p_ = 10^3.48 ± 0.04^·(*R*/[AU])^−1.43 ± 0.07^, (17)

where *R* is in AU and energy in km^2^·s^−2^ (setting proton mass to 1).

Since *E*_t_∝*R*^−1.43^, hence, *dE*_t_/*dR*∝*R*^−2.43^. We also consider that the polytropic index has a slow radial variation, denoted by *γ_a_*− *γ* = *δ_γ_*(*R*); then, from Equation (2) we find *T*_p_ ∝ *R*^−2(^*^γ^*^− 1)^ = *R*^−1.33 − 2^*^δ^*^(*R*)^, and from Equation (7) we have *dq*/*dR*∝*δ_γ_*(*R*)·*R*^−2.33 − 2^*^δ^*^(*R*)^; for slow radial variation, the two results are consistent, both exhibiting a radial gradient *dq*/*dR*∝*dE*_t_/*dR*∝*R*^−x^, with x ≈ 2.33–2.43 (exact match for *δ_γ_* ≈ 0.05).

In another approach, the inner heliospheric plasma is characterized by a variation of the polytropic index from its adiabatic value with *δ_γ_* ~ 0.2, that is, from a near adiabatic index *γ* ~ 1.65 (e.g., [9]) to some smaller value, e.g., *γ* ~ 1.45 (e.g., as derived from [16]); also, see Appendix A. Setting *δ_γ_* ~ 0.2 in Equation (15b), we derive the radial profile of the solar wind heating rate (per proton mass) in units (km^2^/s^3^), depending on the advection speed (as shown in Equation (14)):

(18) $$\frac{1}{m_{p}}\dot{q}(R)/[{\mathrm{km}}^{2}{/s}^{3}]=\left\{ \begin{array}{l} 1.140\cdot 10^{-3}\cdot {\left(R/[\mathrm{AU}]\right)}^{-1.823}\cdot \frac{1+2.383\cdot 10^{-3}\cdot {\left(R/[\mathrm{AU}]\right)}^{2.64}}{1+5.847\cdot 10^{-4}\cdot {\left(R/[\mathrm{AU}]\right)}^{1.22}}\\ 8.946\cdot 10^{-5}\cdot {\left(R/[\mathrm{AU}]\right)}^{2.11}\cdot \frac{1+2.383\cdot 10^{-3}\cdot {\left(R/[\mathrm{AU}]\right)}^{2.64}}{1+5.847\cdot 10^{-4}\cdot {\left(R/[\mathrm{AU}]\right)}^{1.22}}\\ 8.946\cdot 10^{-5}\cdot {\left(R/[\mathrm{AU}]\right)}^{2.11}\cdot {\left[\frac{1+2.383\cdot 10^{-3}\cdot {\left(R/[\mathrm{AU}]\right)}^{2.64}}{1+5.847\cdot 10^{-4}\cdot {\left(R/[\mathrm{AU}]\right)}^{1.22}}\right]}^{\frac{3}{2}}. \end{array}\right.$$

**Figure 1:**
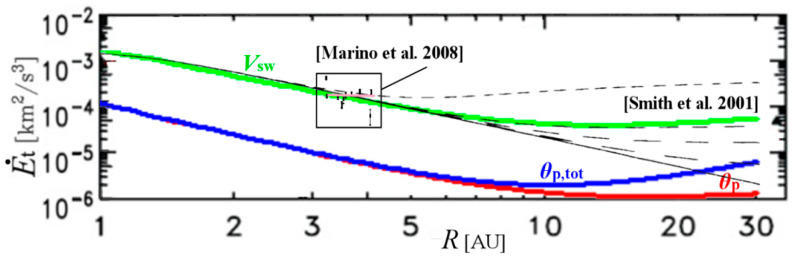
The heat rate $\dot{q}(R)$ is plotted for advection speeds *u*_R_: *V*_sw_ (green), *θ*_p_ (red), and *θ*_p,tot_ (blue), and co-plotted with the modeled (black) values of the turbulent energy rate ${\dot{E}}_{t}(R)$, for the Voyager 2 data computed and plotted as a function of *R* by [29]; the horizontal dash lines represent the modeled turbulent energy rate with adding the effect of PUIs (various models). We observe that the advection speed coincides with the solar wind speed (green) for *R* up to ~10 AU, while it may be better described by the total (solar wind plus pick-up) proton thermal speed (blue) for *R* near ~20 AU and beyond. (Note: all energies are plotted per proton mass). The square inset includes the measurements of turbulent energy transfer rates per *R*, calculated by Marino et al. (Table 1 in [20]). (Note: this figure uses a modified part of Figure 7 from [29].)

In Figure 1, the heat rate $\dot{q}(R)$ (per *m*_p_) is plotted for advection speeds *u*_R_: *V*_sw_ (green), *θ*_p_ (red), and *θ*_p,tot_ (blue). For comparison, we co-plotted the radial profile of the modeled (thin black lines) rate of turbulent energy ${\dot{E}}_{t}(R)$ (per *m*_p_), derived using data from Voyager 2 S/C for *R* between 1 AU–30 AU by [29] (see also [30]). We observe the coincidence of the plotted and modeled turbulent energy with the heat rate up to *R* ~ 10 AU for advection speed given by the solar wind speed (green). Near *R* ~ 20 AU and beyond, the turbulent energy appears to better coincide with the heat rate for advection speed given by the total (solar wind plus pick-up) proton thermal speed (blue).

In addition, the heating rate profile is in agreement with the observational results derived by Marino et al. [20]. In particular, these authors estimated for the first time the turbulent energy transfer rate, which can contribute to the in-situ heating of solar wind. Their results (c.f., Table 1 in [20]) are co-plotted within the square inset in Figure 1, with average energy flux ~(1.86 ± 0.95) × 10^−4^ km^2^/s^3^, ranging between 3.1 AU and 4.2 AU (plotted in the pink horizontal line). The agreement of these measurements with the rest of the modeled graphs is apparent.

In the outer heliosphere, the excitation of plasma waves by newborn interstellar PUIs is the primary source of turbulent heating. The rate of this turbulent energy *dE*_t_/*dt* increases with increasing the heliocentric distance *R* (c.f., Figure 5 in [30]), and is expected to match the heating rate *dq/dt* in Equation (15b). According to this, the rate is proportional to the polytropic difference (*γ_a_*– *γ*), speed, and temperature, and inversely proportional to *R*. Speed decreases slowly with increasing *R*, thus the only way the heating rate can increase with *R* is when the difference (*γ_a_*– *γ*) and/or temperature *T* increase with increasing *R*. Voyager 2 data showed that the temperature increases with *R* beyond some minimum located between 20 AU and 50 AU (c.f., Figure 3 in [30]). In addition, Elliott et al. [14] analyzed New Horizons data for the radial range 22–38 AU. The polytropic index *γ* was derived from fitting eight consecutive pairs of temperature−density values and then averaging the results over solar wind speed for each radial 1 AU-bin of New Horizons data (according to the method of [31]). They found that *γ* decreases, or (*γ_a_*– *γ*) increases, with increasing *R*; specifically, the linear fit to the radial profile of *γ* led to the relationship *γ*(*R*) − 1 ≈ −0.0316·(*R*-30), thus the solar wind is isothermal for *R* near *R*_m_ ~ 30-35AU, while for *R* > *R*_m_ we observed *γ* < 1, corresponding to anti-correlation between *n* and *T* (because of *T* ∝ *n^γ^*^−1^).

Furthermore, we used the relationship of the polytropic index expressed in terms of the heliocentric distance *R* in the outer heliosphere, derived by [14]:

*δ_γ_*(*R*) *≡γ_a_*− *γ*(*R*) = 0.0316·[(*R*/[AU]) − 9], (19a)

in order to derive the heating rate, according to Equation (15b), namely:

(19b) $$\begin{array}{l} \frac{1}{m_{p}}\dot{q}(R)/[{\mathrm{km}}^{2}{/s}^{3}]=\\ \left\{ \begin{array}{l} 5.122\cdot 10^{-2}\cdot [(R/[\mathrm{AU}])-9]\cdot {\left(R/[\mathrm{AU}]\right)}^{-1.823}\cdot \frac{1+2.383\cdot 10^{-3}\cdot {\left(R/[\mathrm{AU}]\right)}^{2.64}}{1+5.847\cdot 10^{-4}\cdot {\left(R/[\mathrm{AU}]\right)}^{1.22}}\\ 4.020\cdot 10^{-3}\cdot [(R/[\mathrm{AU}])-9]\cdot {\left(R/[\mathrm{AU}]\right)}^{2.11}\cdot \frac{1+2.383\cdot 10^{-3}\cdot {\left(R/[\mathrm{AU}]\right)}^{2.64}}{1+5.847\cdot 10^{-4}\cdot {\left(R/[\mathrm{AU}]\right)}^{1.22}}\\ 4.020\cdot 10^{-3}\cdot [(R/[\mathrm{AU}])-9]\cdot {\left(R/[\mathrm{AU}]\right)}^{2.11}\cdot {\left[\frac{1+2.383\cdot 10^{-3}\cdot {\left(R/[\mathrm{AU}]\right)}^{2.64}}{1+5.847\cdot 10^{-4}\cdot {\left(R/[\mathrm{AU}]\right)}^{1.22}}\right]}^{\frac{3}{2}}, \end{array}\right. \end{array}$$

depending on the advection speed, whether it is given by the solar wind flow speed, proton thermal speed, or the solar wind and pick-up proton total thermal speed, as shown in Equation (14).

**Figure 2:**
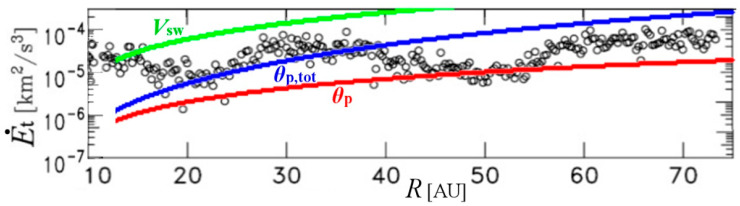
The heat rate $\dot{q}(R)$ is plotted for advection speeds *u*_R_: *V*_sw_ (green), *θ*_p_ (red), and *θ*_p,tot_ (blue), and co-plotted with the turbulent energy rate, ${\dot{E}}_{t}(R)$, originated from the excitation of plasma waves by newborn interstellar PUIs (black circles), which was derived using data from Voyager 2 S/C by [30]; (all energies are plotted per proton mass). The turbulence energy in the outer heliosphere is mostly caused by the scattering of ionized interstellar neutrals (pickup ions), which supplies energy through wave-particle interactions. We observe that the advection speed coincides better with the total (solar wind plus pick-up) proton thermal speed (blue) for *R* near ~20 AU and beyond. (Note: this figure uses a modified part of Figure 5 from [30].).

In Figure 2, the heat rate $\dot{q}(R)$ is again plotted for advection speeds *u*_R_: *V*_sw_ (green), *θ*_p_ (red), and *θ*_p,tot_ (blue). For comparison, we co-plotted the radial profile of the observed (black circles) rate of turbulent energy ${\dot{E}}_{t}(R)$, derived using data from Voyager 2 S/C and computed for *R* between 10 AU and 75 AU by [30]. Beyond 10 AU pickup ions are the dominant source of energy injection into the flow that leads to the heating the solar wind thermal protons. As a result, the advection speed coincides better with the total (solar wind plus pick-up) proton thermal speed (blue) for *R* near ~20 AU and beyond.

The matching of the advection speed with the thermal speed may be explained by the following reasons:

(i) The derivation of energy rate by [30] may not describe the whole PUI turbulent energy;
(ii) The PUI turbulent energy measurements used Voyager 2 dataset [24], while the PUI turbulent energy modeled by this paper used the PUI kinetic energy measured by New Horizons dataset [30]. Moreover, the two datasets were collected on different parts of solar cycle;
(iii) Part of the PUI kinetic energy may be transformed into turbulent energy. In general, the PUI average kinetic energy *Ε*_pui_ is different to the energy from the excitation of plasma waves by newborn interstellar PUIs, *Ε*_t,pui_, that is, one of the sources of solar wind turbulent heating. Newly-born PUIs are created as an unstable ring beam in the plasma frame, generating magnetic waves that pitch angle scatter them. Τhe process of PUI isotropization (pitch angle scattering to near-isotropy) yields heating of the solar wind ions via energy transfer with magnetic wave interactions. The energy that is created into magnetic waves is a small fraction of the gyrational energy of the PUIs, *E*_pui_. Indeed, *E*_pui_ is significantly larger than *Ε*_t,pui_; e.g., at *R* ~ 40AU, the rate of *E*_pui_ is ~1.5 orders of magnitude larger than the rate of *Ε*_t,pui_:
  *dΕ*_t,pui_/*dR* ≈ 8·[km/s]^2^·[AU]^−1^ and *dE*_pui_/*dR* ≈ 245·[km/s]^2^·[AU]^−1^ (per proton mass). (20)
  (Note: for *Ε*_pui_, see Figure 7 in [24]; for *E*_t,pui_ see the rate of change in Figure 5 in [30], which is co-plotted in Figure 2;)
(iv) More complicated modeling of the heat flux may be necessary, e.g., including proton and electron species [31];
(v) Finally, advection at thermal speed, instead of the bulk flow speed, is possible as a limiting case, e.g., see: [21,22,23].

Moreover, in Figure 2 we observe a variation between the curves representing advection at the total proton or at just the solar wind proton thermal speeds. It is possible that the advection occurs simply via the solar wind protons, thus, at their thermal speed. Then, as the solar wind expands farther in the outer heliosphere, the intensity and effect of pick-up ions increases, while the already picked-up protons have become matured enough to also contribute to the advection; hence, the shift to the total thermal speed as *R* increases beyond ~50 AU. Surely, future analyses will reveal the quantitative details of this process.

## 5. Conclusions

The paper improved the understanding of the interplay and partition of the sources of proton plasma turbulent heating of the expanding solar wind in the inner and outer heliosphere.

The sources of turbulence in the heliosphere can be divided into two groups: (1) solar-origin large-scale energy fluctuations (stream shears and shock waves) driven turbulence, and (2) interstellar PUI driven turbulence (e.g., [27]). Both of these groups of sources contribute to solar wind heating, but (1) is dominant in the inner heliosphere and (2) is dominant in the outer heliosphere.

The turbulent energy from solar-origin sources is decreasing along the solar wind radial expansion in the heliosphere; however, the turbulent energy from the interstellar-PUI-origin source is increasing (denoted with *Ε*_t,pui_). See, for example, the radial profile of the rate of *Ε*_t,pui_ in the outer heliosphere, which increases with increasing *R*, estimated using Voyager 2 data by [30].

We showed the connection between the polytropic index and the turbulent energy rate in the solar wind proton plasma, by comparing their corresponding radial profiles in the outer and inner heliosphere.

Specifically, we observed that the advection speed coincides with the solar wind speed (green) for *R* up to ~10 AU, while it better coincides with the total (solar wind plus pick-up) proton thermal speed (blue) for *R* near ~20 AU and beyond.

In summary, we:

(i) Showed the theoretical connection of the rate of a heat source, such as the turbulent energy, with the polytropic index and the thermodynamic process;
(ii) Calculated the effect of the pick-up protons in the total proton temperature and the relationship connecting the rate of heating with the polytropic index;
(iii) Derived the radial profiles of the solar wind heating in the outer and inner heliosphere;
(iv) Used the radial profile of the turbulent energy in the solar wind proton plasma in the outer and inner heliosphere, in order to show their connection with the radial profiles of the polytropic index and the heating of the solar wind.

While we showed the first evidence of the connection among the polytropic index *γ*, the rate of the heat absorbed by the solar wind *dq/dt*, and the rate of turbulent energy *dE_t_^+^/dt*, during the heating of the solar wind in the inner and outer heliosphere, the exact connection between them is unknown.

Future data and theoretical analyses can determine the Elsässer variables, *E_t_^+^* and *E_t_^−^*, and employ the theoretical 3D-model [30] to model the evolution of the turbulent energy, and then compare with the heating related to the polytropic index. Datasets of both the inner (e.g., Helios 1 and 2, ACE, Wind, Ulysses) and outer (Voyager 1 and 2, updated New Horizons, Pioneers 1 and 2) heliosphere can be used. Furthermore, the analysis of Elliott et al. [14] can be repeated to derive the polytropic index radial profile using Voyager 1 and 2 datasets, and compare it with the results of [14] for the New Horizons data. The connection of the polytropic index with (i) the kappa index [12,32], the parameter that governs and labels kappa distributions [33,34], (ii) the Debye length [35,36,37,38,39], (iii) the mean-free-path [40], may be used and involved.

## Appendix A

In the appendix, we summarize the formulae and method used for the derivation of the polytropic index, its smoothing averaged value and its standard error. (For more details, see: [11].)

We use the density *n* and temperature *T* of the *i*th and (*i* + 1)th data points to derive the polytropic index at the *i*th data point and its error, that is:

(A1a) $$\gamma_{i}=1+\ln(T_{i+1}/T_{i})/\ln(n_{i+1}/n_{i}),$$

(A1b) $$\delta \gamma_{i}=\sqrt{2}\cdot \sqrt{{\left(\delta \ln T\right)}^{2}+{\left(\gamma_{i}-1\right)}^{2}{\left(\delta \ln n\right)}^{2}}\cdot {\left[\ln(n_{i+1}/n_{i})\right]}^{-1}.$$

Note: we apply the above when *i*th and (*i* + 1)th data points correspond to invariant Bernoulli integral to reduce the possibility of streamline crossing [5,7].

According to [9], smoothing the time series involves finding the *M-*step moving average:

(A2) $$\bar{\gamma }(M)=\sum_{i=1}^{M}\delta \gamma_{i}{}^{-2}\gamma_{i}/\sum_{i=1}^{M}\delta \gamma_{i}{}^{-2}.$$

The error of the weighted mean is a combination of the propagated uncertainty:

(A3a) $$\delta {\bar{\gamma }}_{}^{prop}=\frac{1}{\sqrt{\sum_{i=1}^{M}\delta \gamma_{i}^{-2}}},$$

and the statistical uncertainty (which is related to the curvature of the total residuals, e.g., [41,42,43]):

(A3b) $$\delta {\bar{\gamma }}_{}^{stat}=\sqrt{\frac{\frac{1}{M}\sum_{i=1}^{M}\delta \gamma_{i}^{-2}{\left(\gamma_{i}^{}-\bar{\gamma }\right)}^{2}}{\sum_{i=1}^{M}\delta \gamma_{i}^{-2}-{\left(\sum_{i=1}^{M}\delta \gamma_{i}^{-2}\right)}^{-1}\cdot \sum_{i=1}^{M}\delta \gamma_{i}^{-4}}},$$

which are combined to give the total uncertainty of the weighted mean:

(A3c) $$\delta \bar{\gamma }(M)=\sqrt{{\left(\delta {\bar{\gamma }}_{}^{prop}\right)}^{2}+{\left(\delta {\bar{\gamma }}_{}^{stat}\right)}^{2}}.$$

(For several applications of the above statistics, see: [41,42,43,44,45,46,47].)

The above were applied in [9], showing that the polytropic index uncertainty is of the order ~0.2 in the solar wind proton plasma near 1 AU (c.f., the mean and standard error of the polytropic indices plotted in Figure 4 and Figure 5 in [9]).
